# Heavy metal and metalloid concentrations in red deer (*Cervus elaphus*) and their human health implications from One Health perspective

**DOI:** 10.1007/s10653-024-01991-8

**Published:** 2024-06-07

**Authors:** Catarina Jota Baptista, Fernanda Seixas, José M. Gonzalo-Orden, Carla Patinha, Pedro Pato, Eduardo Ferreira da Silva, Gilberto Fernandes, Paula A. Oliveira

**Affiliations:** 1 Departamento de Ciências Veterinárias, Escola de Ciências Agrárias E Veterinárias (ECAV), Universidade de Trás-Os-Montes E Alto Douro (UTAD), Vila Real, Portugal; 2 Centro de Investigação das Tecnologias Agroambientais E Biológicas (CITAB/ Inov4Agro), UTAD, Vila Real, Portugal; 3 Instituto de Biomedicina (IBIOMED), Universidad de León, León, Spain; 4 Egas Moniz Center for Interdisciplinary Research (CiiEM), Egas Moniz School of Health & Science, Almada, Portugal; 5 Centro de Ciência Animal E Veterinária (CECAV), Associated Laboratory for Animal and Veterinary Sciences (AL4AnimalS), UTAD, Quinta de Prados, 5000-801 Vila Real, Portugal; 6 GEOBIOTEC and Departamento de Geociências, Universidade de Aveiro, Aveiro, Portugal

**Keywords:** Metal(loid), One Health, Pathology, Pollution, Trace element, Ungulate

## Abstract

The red deer is an ungulate and large game species. The contamination of the ecosystems by metal(loid)s may lead to the exposure of animals (as well as humans) through water and food resources. The direct contact of hunters and wild animal meat consumers with deer carcasses may be a potential contaminant source. This study aimed to determine the metal(loid)s’ concentrations in the liver and kidney of red deer from two regions of Portugal (Idanha-a-Nova and Lousã), and to relate these with histopathologic lesions. Thirteen young male deer were submitted to metal(loid) determination (As, Cd, Co, Cr, Cu, Pb, and Zn) by inductively coupled plasma mass spectrophotometry (ICP-MS) and histopathology examination. Renal Cd (8.072 ± 5.766 mg/kg dw) and hepatic Pb (3.824 ± 6.098 mg/kg dw) mean values were high, considering the maximum values for consumption established by the European Commission. The hepatic mean value of Cu was significantly higher in Idanha-a-Nova (150.059 ± 33.321 mg/kg dw), and it is at the Cu toxicity limit considered for ruminants (150 mg/kg). The pollution induced by Panasqueira mines (Castelo Branco) may be a possible explanation for some of the findings, especially the higher values of hepatic Cu and Pb found in Idanha-a-Nova deer. These results have high importance under a One Health perspective, since they have implications in public health, and pose at risk the imbalance of animal populations and ecosystems.

**Supplementary Information:**

The online version contains supplementary material available at 10.1007/s10653-024-01991-8.

## Introduction

The red deer (*Cervus elaphus*) is a wild ungulate, widely distributed in the Iberian Peninsula and other European countries (Mattioli et al., 2022). Hunting and game meat consumption represent a vital part of the economy of most European countries (Fantechi et al., 2022).

As a game species, the red deer (and other deer and wild boars) are in direct contact with hunters and wild meat consumers, serving as a potential source of pathogens and contaminants, not to mention their share of land and water resources with domestic species. Therefore, wild populations should be constantly and carefully monitored for distinct hazards to avoid potential One Health consequences. Under a One Health approach, it is assumed that human health is closely related to animal and environmental health, as three interconnected branches. One Health principles have been more used and relevant in the past few decades, although it is not new (Centers of Disease Control and Prevention (CDC), 2022; Hryhorczuk et al., 2018). Environmental pollution is, therefore, a fundamental One Health concern. From a public health point of view, some of the most critical chemical pollutants include organic pollutants, drugs, and inorganic contaminants (such as heavy metals and metalloids) (Buttke, 2011; Hryhorczuk et al., 2018; Kendall et al., 2010).

Heavy metal(loid)s (also known as trace elements) can be considered essential (as Cr, Cu or Zn) and non-essential (as As, Cd or Pb), even though both can be toxic to a living being (including humans), depending on several factors, such as the frequency of exposure, dose, or susceptibility factors. Essential metal(loid)s have a biological function, whereas non-essential have no biological function. Metal(loid)s may provoke acute clinical lesions in a living being or accumulate during the entire life, leading to discrete and chronic effects in an individual or population (e.g. reproductive indicators, population imbalances, among others) (Ali & Khan, 2019; Ali et al., 2019; Castellanos et al., 2010; Levengood & Heske, 2008). Histopathological analysis can be performed in parallel with the metal(loid)s determinations to access lesions and organic effects of these compounds (McNamara, 2016), and the liver and kidney are the most used organs due to their role in the metabolism and excretion of xenobiotics (Jota Baptista et al., 2022). Previous studies in Europe have been assessing heavy metal(loid)s in several wild mammals. Game species have been frequently used due to their general accessibility, distribution and the direct impact in the health of consumers. However, there is a lack of studies and continuous monitoring of these populations in the Iberian Peninsula, especially considering their abundance, proximity to humans and livestock, consumption habits and economical importance in these countries (Hampton et al., 2023; Jota Baptista et al., 2023a, 2023b).

The aims of the present study are (1) to determine the metal(loid)s’ concentrations in liver and kidney tissues of hunted red deer from two regions of Portugal (Idanha-a-Nova and Lousã), (2) to relate these metal(loid) concentrations with histopathologic lesions, and (3) to raise awareness for possible potential public health implications linked the consumption of red deer.

## Methods

### Sampling

Thirteen deer killed during the hunting season in Portugal, between August to October 2021, were included in this study: nine from Idanha-a-nova (Castelo Branco, Portugal), and four from Serra da Lousã (Coimbra, Portugal) (Supplementary file 1). All these deer were young males with an age range of three to six years-old. None of the deer was killed explicitly for this study, so no ethics approval was required. All the animals were hunted following the national legislation for hunting this large game species.

Idanha-a-Nova is a geographical area of concern for wild ungulate management, mainly due to certain zoonotic infectious diseases, such as tuberculosis. Nevertheless, this region's hunting activities and livestock are relevant economic activities, reinforcing the importance of constant monitoring (Santos et al., 2015; Vieira-Pinto et al., 2011). The Panasqueira mining area is located in Castelo Branco district. This is the oldest mining company of Portugal, in function for more than one hundred years, and has been an important source of wolfram (W) for many years. Other elements found in this geographical area include Ag, As, Bi, Cu, Pb, Sn, and Zn (Ávila et al., 2017; Candeias et al., 2014).

Serra da Lousã is a mountain range in Coimbra District, that includes a natural park with a high density of red deer and no natural predators. Some agricultural activity can also be seen in the park's surrounding areas (Monzón et al., 2012).

The kidney and liver samples were collected just after the deer’ death during the hunting season. Twenty grams of kidney and liver were placed in 10% formalin and maintained at room temperature for histopathology, and ten grams were placed in zip bags and stored at −20 °C for metal(loid) analysis.

### Histopathological analysis

Kidney and liver samples were sent to the Histopathology Laboratory for analysis under blind test in an optical microscope (Nikon E600®). At first, they were submitted to routine histological techniques. Samples were trimmed into small slices (2–3 mm) and placed inside tissue cassettes in the fume hood (GrossLab, Shandon®, Thermo, Waltham, MA, USA). Then, they were dehydrated and embedded in paraffin in a tissue processor (Dakewe HP300®, Shenzhen, China). Paraffin blocks were performed in an embedding station (Histocentre, Shandon®, Thermo, Waltham, MA, USA). Afterward, slides were prepared in an automated microtome (Leica RM2255®, Leica Microsystems, Wetzlar, Germany) and stained with hematoxylin and eosin in a slide stainer (Varistain 24–4, Shandon®, Thermo, Waltham, MA, USA).

### Determination of Metal(loid)s

Twenty-four hours before lyophilisation, samples were maintained under −20 °C. Liver and kidney samples were completely freeze-dried for 48 h under −56 °C (LaboGene CoolSafe®, Allerød, Denmark). The humidity lost by each sample (liver: 51.47%; kidney: 55.22%) was calculated by measuring their weight before and after the freeze-drying procedure on a precision scale (Kern ALT®, Germany).

Nearly 0.5 g of each freeze-dried sample was transferred to acid digestion tubes. 1 mL of nitric acid was added to each sample; the tubes were gently shaken and left at room temperature overnight. Then, 2 mL of hydrogen peroxide was added to each tube, the tubes were manually shaken once again and left for approximately 5 h at room temperature. Then, tubes were placed on a digestion plate (DigiPrep-MS®, Canada), which was programmed to increase the temperature for 15 min until 85 °C progressively. Then, they spent another 15 min under 85 °C. Metal(loid)s determinations were done by inductively coupled plasma mass spectrophotometry (ICP-MS) (Agilent 7700, Agilent Technologies®, Santa Clara, USA).

A quality control procedure was applied to metal(loid)s analysis. Visual inspection of the tubes was conducted after each digestion cycle, and they were all considered correctly digested when no solid particles were visible. Moreover, ERM BB185® (Belgium) was used as a certified reference material, as well as blank tubes and duplicates. Results were considered when recoveries ranged between 70 and 120%. The quantification limits (LOQ) were 0.025 mg/kg for As, 0.01 mg/kg for Cd, Co, Cr, Cu, and Pb, and 0.1 mg/kg for Zn. Values above these were considered zero.

### Statistical methods

IBM® SPSS Statistics 27 was the statistical program used for all the descriptive statistics and statistical tests. Normality tests were applied to metal(loid)s concentrations (Shapiro–Wilk tests and Kolmogorov–Smirnov). For most elements, non-normal distributions were registered. Thus, non-parametric tests (Kruskal–Wallis and Mann–Whitney tests) were used to correlate the metal(loid) concentrations with the geographical provenances (Idanha-a-Nova and Lousã) and histopathology lesions.

For all the statistical tests, a critical *p*-value of 0.05 was considered.

## Results

### Metal(loid)s determinations

The mean and standard deviation (SD) obtained for each element in each organ (liver or kidney) were summarised in Table 1.

**Table 1:** Mean and standard deviation (SD) of each metal(loid) determination, considering the provenance of the deer (mg/kg dry weight [dw])

Organ	Metal(loid)	Total (n = 13)		Idanha-a-Nova (n = 9)		Lousã (n = 4)		ML**
		Mean	SD	Mean	SD	Mean	SD	
Liver	As	0.044	0.013	0.046	0.014	0.040	0.007	–
	Cd	0.370	0.103	0.392	0.101	0.321	0.103	1.030
	Co	0.361	0.158	0.340	0.096	0.409	0.268	–
	Cr	0.081	0.062	0.074	0.069	0.097	0.044	–
	Cu*	132.141	39.028	150.059	33.321	91.828	1.319	–
	Pb	3.824	6.098	5.466	6.776	0.128	0.071	0.412
	Zn*	121.178	36.019	106.857	30.518	153.402	26.573	–
Kidney	As*	0.097	0.036	0.082	0.028	0.133	0.024	–
	Cd	8.072	5.766	5.857	0.925	13.057	9.103	2.233
	Co	0.256	0.114	0.231	0.043	0.313	0.201	–
	Cr	0.156	0.178	0.170	0.203	0.123	0.120	–
	Cu	24.514	4.639	25.439	5.019	22.432	3.250	–
	Pb	0.378	0.491	0.455	0.580	0.207	0.102	0.446
	Zn	216.968	121.423	186.163	84.769	286.279	174.814	–

^*^Metal(loid)s with statistically significant different concentrations between Lousã and Idanha-a-Nova deer populations

^**^ML concentration in mg/kg dry weight, converted according to the calculated humidity loss and the reference value provided in EU regulation No.915/2023 (0.20 mg/kg wet weight [ww] for Pb in both organs, for bovines, 0.5 mg/kg ww for Cd in the liver, and 1.0 mg/kg ww for Cd in kidney, for livestock)

There were no statistically significant differences between the two populations (Lousã and Idanha-a-Nova) for most metal(loid)s. The exceptions were Cu in the liver (*p* = 0.034), Zn in the liver (*p* = 0.020), and As in the kidney (*p* = 0.011). While deer from Idanha-a-Nova presented higher mean values of hepatic Cu, animals from Lousã showed higher mean values of hepatic Zn. Regarding As in the kidneys, deer from Lousã presented higher average levels.

### Histopathology

**Fig. 1:**
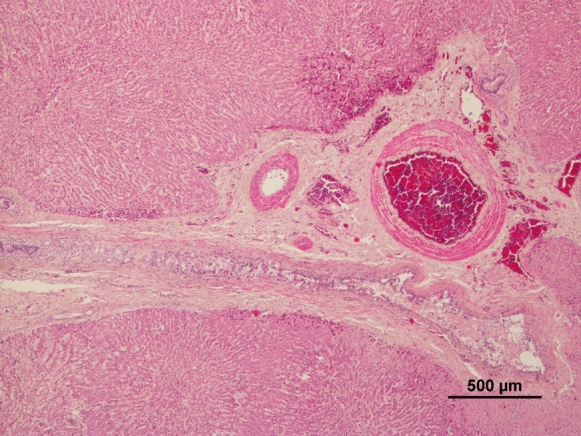
Deer liver with evident portal fibrosis, biliary hypertrophy and hyperplasia

**Fig. 2:**
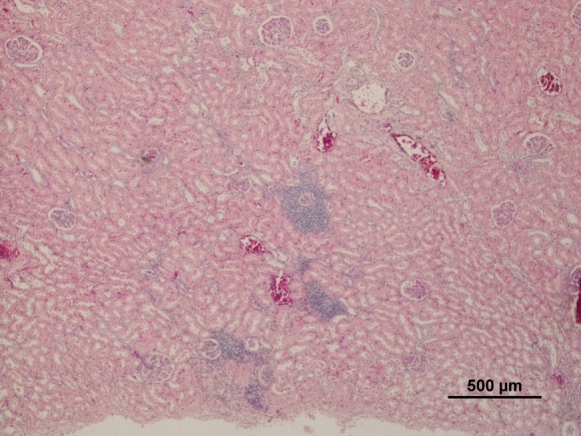
Deer kidney with non-purulent nephritis

Most deer (8/13) presented no significant histopathologic changes in the liver. However, some animals showed portal fibrosis (3/13), biliary hyperplasia (3/13), vacuolar and hydropic changes (3/13) and congestion (4/13) (Fig. 1); one deer showed of eosinophilic infiltration (1/13) probably due to parasite infection. Animals with biliary hyperplasia presented statistically significant higher mean values of Zn (*p* = 0.007). In contrast, compared to the others, they showed lower values of hepatic Pb (*p* = 0.014), Cd, and Co (*p* = 0.007, for both). Regarding the kidney, four animals showed non-purulent nephritis (5/13) (Fig. 2), one presented vascular congestion (1/13). Seven deer presented histologically normal kidneys (7/13).

## Discussion

Thirteen livers and kidneys were submitted to metal(loid) determination and histopathology. Then, these results were interpreted and compared to other findings, mostly in ungulates.

In total, As mean concentrations were 0.044 ± 0.013 mg/kg dw and 0.097 ± 0.036 mg/kg dw in the liver and kidney, respectively. Sources of As to animals may include feed additives and fertilisers, and cases of chronic exposure (and its carcinogenic effects to, for instance, the liver, bladder and lung) are more frequently detected in humans and domestic species than in wildlife (Eisler, 1988). Deer from Lousã presented statistically significant higher values in the kidney (0.133 ± 0.024 mg/kg dw), compared to animals from Idanha-a-Nova (0.082 ± 0.028 mg/kg dw).

Like in other studies and geographical locations (Kottferová & Koréneková, 2000; Pompe-Gotal & Crnić, 2002; Santiago et al., 1998), the mean value of Cd in the kidney (8.072 ± 5.766 mg/kg dw) was very high in the present study. As herbivores, they depend on large amounts of plants, whose roots and leaves tend to accumulate high amounts of Cd (Santiago et al., 1998). Moreover, the detected levels of Cd in the liver (0.370 ± 0.103 mg/kg dw) were much lower than the kidney Cd levels (8.072 ± 5.766 mg/kg dw), i.e. hepatic Cd/renal Cd is substantially lower than 1; which suggests a chronic exposure (rather than acute), since Cd is usually transported from liver to the kidney during the life of these animals (Santiago et al., 1998). Furthermore, differences in the plant availability and selection may be responsible for a great variance in the amount of Cd detected in the offal (Dudley et al., 1985; Ríos C. & Méndez-Armenta, 2019).

According to the Commission Regulation (EC No. 915/2023) and considering the humidity of the analysed tissues, a maximum concentration of 1.030 mg/kg dw (0.50 mg/kg ww) for Cd, and 0.412 mg/kg dw (0.20 mg/kg ww) for Pb would be allowed in a consumed liver. The mean values of hepatic Pb are above this, considering the total number of deer (3.824 ± 6.098 mg/kg dw), but especially in Idanha-a-Nova (5.466 ± 6.776 mg/kg dw). Considering the importance of hunting and livestock activities in Idanha-a-Nova, these results should raise awareness of hunters and public health agents, and potentially indicate high contamination of Pb in the soils and water used by these animals. One possible source of contamination in this region might be the Panasqueira mines, located nearby (Ávila et al., 2017; Candeias et al., 2014, 2015, 2020; Ferreira, 2004). Nevertheless, these maximum values have been calculated for domestic species and not for game species, which are normally less frequently consumed by the population. These Pb mean values (with a high variation) should be interpreted with caution, but always considering the accumulation properties of this substance and its chronic effects (neurotoxic, cardiotoxic, nephrotoxic, and hepatotoxic), especially for children (Hampton et al., 2023). On the other hand, according to a recent fact sheet launched by the World Health Organization (WHO), “there is no level of exposure to lead that is known to be without harmful effects” (Hampton et al., 2023; World Health Organization, 2023). Therefore, Pb exposure should always be assumed to have dangerous health effects, regardless of the dose or frequency of exposure.

Concentrations of 2.233 mg/kg dw (1.0 mg/kg ww), and 0.446 mg/kg dw (0.20 mg/kg ww) are permitted for Cd, and Pb, respectively, are permitted for the kidney (Commission Regulation, 2023). The variation (SD) is extensive, but the kidney values of Cd in the present study were generally elevated (8.072 ± 5.766 mg/kg dw), especially for Lousã deer (13.057 ± 9.103 mg/kg dw). Once again, these results should be interpreted carefully, although some awareness should be raised for future detailed studies. It is not advisable to consume kidneys from game species, and it is generally discarded during dismantling or preparation of the carcass with other offal.Notwithstanding, contamination of other tissues may happen during these procedures, not to mention the persistency of this element in surfaces, instruments, or dumping sites (especially if the offal is inappropriately discarded in the environment) (Ertl et al., 2016; Hampton et al., 2023; Kottferová & Koréneková, 2000).

Considering Co, Cr, Cu, and Zn (all essential elements), their interpretation should take into account that, rather than non-essential metal(loid)s, they all have a biological function (i.e. they are desirable for good nutrition of the animals). Thus, maximum tolerable limits usually are higher than for non-essential elements. The composition of the pasture has a strong influence on the assimilation of these micronutrients, in their internal values and, consequently, in a final game meat product (Soriano & Sánchez-García, 2021). In general, Zn and Cu are usually abundant, followed by Cr and Co, as observed in the present study. A study on white-tailed deer (*Odocoileus virginianus*) in Illinois (USA) (*n* = 190) reported higher mean hepatic values of Cr (2.7 ± 0.1 mg/kg dw), but lower Co (0.18 ± 0.01 mg/kg dw), Cu (109 ± 5 mg/kg dw), and Zn (70 ± 2 mg/kg dw) (Woolf et al., 1982). According to the literature, hepatic levels of Cu above 150 mg/kg dw have been associated with toxicity in ruminants (Buck et al., 1976). However, different species may present distinct adaptative mechanisms to the negative effects of xenobiotics. The variability of metal(loid)s concentrations found among different cervids with no signs of toxicity (or deficiency) possibly reflects species adaptations (Vikøren et al., 2011). Deer from Lousã present a mean value of hepatic Cu below this limit (91.828 ± 1.319 mg/kg dw), but the deer from Idanha-a-Nova presented a statistically significant higher value that was precisely at this toxicity limit (150.059 ± 33.321 mg/kg dw; *p* = 0.034).

Some of the detected alterations (as vacuolar lesions and congestion) may indicate an initial phase of hepatic reversal lesion and oxidative stress of hepatic cells. Several studies showed that metal(loid)s exposure promotes radicals of oxidative stress (ROS) production, and ROS promotes the production of peroxides, which contribute to cell membrane damage (Kim et al., 2021). Some elements, such as Cu and Pb, are frequently associated with hepatic severe lesions. Since our deer were young males (three- to six-year-old; life expectancy 12–13 years), the chronic contact with metal(loid)s may not had been long enough to produce irreversible lesions (with cell necrosis and hepatic failure), but sufficiently long to induce those reversible alterations (Lazarus et al., 2008; Vikøren et al., 2011). Nevertheless, these lesions may have other origin rather than toxic, such as infectious or nutritional. Future assessments are welcome, using deer from a control and a polluted area for comparison, and measuring biochemical biomarkers (e.g. lipid peroxidation or oxidative cell stress biomarkers, live enzymes and renal function analysis as urea and creatinine), which are usually more specific for these lesions (Hampel et al., 2016; Laguna-Ruíz et al., 2001; Tête et al., 2015). The absence of severe histopathologic lesions may be interpreted as a good indicator, it seems that the health of these animals is not posed at severe risk. However, it is not possible to guarantee the same for older deer, exposed to these pollutants for a longer time. It also reinforces that heavy metal(loid)s chronical pollution is usually an inconspicuous problem (Dahiya, 2022; Nriagu, 1988), and it may be challenging to detect by visual inspection methods of deer carcasses in the field. New studies assessing liver and kidney function biomarkers should be performed to clarify early effects of metal(loid)s overload on liver and kidney, in association with the metal(loid) concentrations.

Despite their harmful effects on health, Cd, and Pb are considered the most frequent metallic contaminants of the Mediterranean forest (Santiago et al., 1998), which also supports the concerning values found in the present study, as well as the need for future and continuous assessments of the fauna and ecosystems, especially fauna and flora species consumed by humans. Moreover, it would be very relevant to evaluate the risk of consumption specifically for game meat products and, therefore, establish specific maximum levels for these, at a national or European level, considering everyday consumption habits. This would undoubtedly provide helpful and practical information to hunters, consumers and public health professionals and help define risk regions.

The mean values of renal Cd (8.072 ± 5.766 mg/kg dw) and hepatic Pb (3.824 ± 6.098 mg/kg dw) were high and should be noted for future assessments, even considering that histopathology did not show severe toxic effects of metal(loid)s exposure. The levels of essential elements were in line with other studies. The higher values of hepatic Cu and Pb in Idanha-a-Nova reinforce the need to monitor wildlife that lives near mining (and other potentially contaminated) areas, especially species of consumption. Some of the tissue lesions (as vacuolar changes and congestion) may indicate an initial reversal phase of response to this high metal(loid)s values.

Notwithstanding, the low number of samples and short collection period analysed during this study should be mentioned as study’ limitations. The authors of the present work believe that further conclusions can be taken with more complete and long-term studies using a higher sample size, as well as biochemical biomarkers of exposure.

## Conclusions

The metal(loid)s pollution of the ecosystems, and the consequent contamination of food products could be interpreted as a severe but silent threat to One Health, posing at risk the imbalance of animal populations and public health in a discrete way. These results should not cause panic or immediately call into question the consumption of these food products. However, they certainly support the brief creation of the following measures: (1) evaluate the specific risk of regular consumption of game meat, considering inorganic pollutants; (2) create a specific maximum level for wild meat and offal (for As, Cd, Pb and also including essential elements); (3) educate hunters and consumers for this public health hazard (as it has been done for zoonotic infectious diseases); (4) design a continuous monitoring program of this issue at national or European level, defining regions of priority of each contaminant (considering possible sources of pollution, such as the Panasqueira mines). Further research (including animals from different areas of Portugal; or using other tissues as muscle) is necessary to establish which zones should be prioritised for each metal(loid) and apply mitigation strategies (for instance, phytoremediation).

### Supplementary Information

Below is the link to the electronic supplementary material.

Supplementary file1 (PDF 1375 kb)

## Data Availability

There is no data available from this study.
