# Supplementary material for: Heavy metal and metalloid concentrations in red deer (Cervus elaphus) and their human health implications from One Health perspective
Source: Environ Geochem Health. 2024 Jun 7;46(7):226. doi: 10.1007/s10653-024-01991-8 (PMC11161529; doi:10.1007/s10653-024-01991-8)
Supplement: Supplementary file 1 — Supplementary file1 (PDF 1375 kb) [file 10653_2024_1991_MOESM1_ESM.pdf]

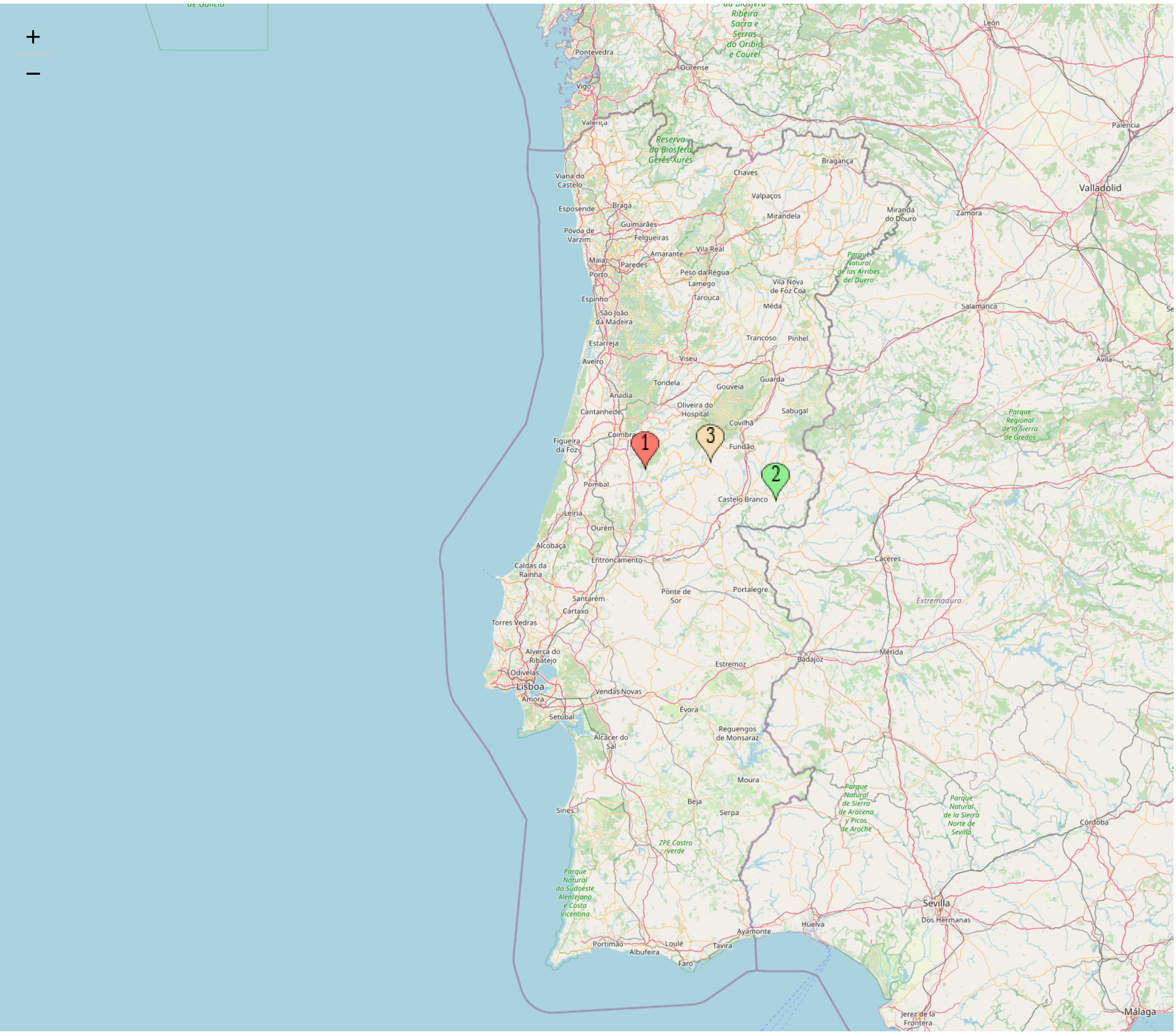

Leaflet (https://leafletjs.com) | Map data © OpenStreetMap (https://www.openstreetmap.org/) contributors, ODbL (https://opendatacommons.org/licenses/odbl/1.0/)

1		Lousã, Coimbra, Portugal
2		Idanha-a-Nova, Castelo Branco, Portugal
3	Panasqueira mines, Castelo Branco	Minas da Panasqueira, Rua do Clube Desportivo, Barroca Grande, Aldeia de São Francisco de Assis, Covilhã, Castelo Branco, Portugal
